# Phosphoproteome Discovery in Human Biological Fluids

**DOI:** 10.3390/proteomes4040037

**Published:** 2016-12-01

**Authors:** Francesco Giorgianni, Sarka Beranova-Giorgianni

**Affiliations:** Department of Pharmaceutical Sciences, University of Tennessee Health Science Center, Memphis, TN 38163, USA; fgiorgia@uthsc.edu

**Keywords:** phosphoproteome, mass spectrometry, biological fluid, biomarker

## Abstract

Phosphorylation plays a critical role in regulating protein function and thus influences a vast spectrum of cellular processes. With the advent of modern bioanalytical technologies, examination of protein phosphorylation on a global scale has become one of the major research areas. Phosphoproteins are found in biological fluids and interrogation of the phosphoproteome in biological fluids presents an exciting opportunity for discoveries that hold great potential for novel mechanistic insights into protein function in health and disease, and for translation to improved diagnostic and therapeutic approaches for the clinical setting. This review focuses on phosphoproteome discovery in selected human biological fluids: serum/plasma, urine, cerebrospinal fluid, saliva, and bronchoalveolar lavage fluid. Bioanalytical workflows pertinent to phosphoproteomics of biological fluids are discussed with emphasis on mass spectrometry-based approaches, and summaries of studies on phosphoproteome discovery in major fluids are presented.

## 1. Introduction

Phosphorylation is a common post-translational modification of proteins that involves the reversible attachment of phosphate groups to the side chains of specific amino acids. *O*-phosphorylation occurs most commonly on serine (Ser) and threonine (Thr) residues; a small fraction of phosphorylation (less than 1%) is present on tyrosines (Tyr) [1]. The human phosphoproteome is a highly complex and dynamic system. One-third of human proteins are phosphorylated, often at multiple sites and in a transient manner, with phosphorylation–dephosphorylation events orchestrated by an array of kinases and phosphatases [2].

Phosphorylation serves as a means to fine-tune protein function and it participates in virtually all cellular processes. Aberrations in protein phosphorylation have been linked to a wide variety of human diseases including cancer [3,4], heart disease [5,6], obesity and diabetes [7,8], and neurodegenerative diseases [9]. With the advent of modern, high-throughput bioanalytical technologies and bioinformatics tools and resources, examination of protein phosphorylation on a large scale has been enabled, and phosphoproteomics has become a major area in biomedical research.

Biological fluids are relatively easily accessible specimens that have been a longstanding focus of proteomics research, primarily in the context of discovery and development of new biomarkers for diagnosis of a disease, for evaluation of disease progression, or for selection of targeted therapies and monitoring of therapeutic effectiveness. The initial excitement that drove expansion of biomarker discovery proteomics as one of the main thrusts in the post-genome era has been somewhat dampened by the much-slower-than-expected progress in translating these efforts into improvements in clinical practice. The proteomics community is taking a critical look at the reasons behind the disappointing rate of translation of the “proteomics promise” to identify key problem issues and to devise strategies how to overcome them [10]. Expanding beyond measurement of alterations in protein levels, an increasingly prominent branch of biological fluid proteomics focuses on analysis of post-translational modifications, including the glycoproteome [11] and the phosphoproteome.

The biological fluid phosphoproteomes include phosphoproteins secreted or shed from cells, and those entering the fluids from leakage of intracellular content from damaged cells. Furthermore, increasing evidence indicates that proteins may be phosphorylated in extracellular spaces through actions of ectokinases [12,13].

Serum/plasma reflects the status of distant tissues, which collectively contribute to the overall (phospho)proteome profile. Proximal fluids such as cerebrospinal fluid (CSF) or bronchoalveolar lavage fluid (BAL) reflect more specifically the health/disease processes of the particular organ(s); these fluids are more likely to contain higher concentrations of organ-specific marker proteins and therefore provide a more direct molecular readout of the local milieu from which they originate. Whether through analysis of serum/plasma as the broadest survey of body physiology or through examination of proximal fluids for focused assessment of specific tissues/organs, it is envisioned that elucidation of disease-specific alterations in phosphorylation profiles will bring new ways for early detection and diagnosis of diseases, and for tailoring of therapy. Furthermore, information on phosphoproteome dynamics in biological fluids could be utilized towards novel mechanistic insights via integration with other types of molecular data using systems biology tools and approaches [14]. Defining the biofluid phosphoproteome may also be the initial step to targeted functional examination of specific proteins/sites, including the kinases that phosphorylate these sites.

From the bioanalytical standpoint, large-scale analysis of complex phosphoproteomes is a highly challenging endeavor. During the last decade, tandem mass spectrometry (MS/MS) has emerged as the principal technology for global-scale qualitative and quantitative examinations of protein phosphorylation. Analogously to phosphoproteome investigations in cells and tissue, MS/MS plays a critical role in bioanalytical strategies applied to analyses of the phosphoproteome in biological fluids, owing to its sensitivity, throughput, and the capability to provide information on protein identity as well as on the precise location of the site(s) of phosphorylation. Workflows for MS/MS-centric phosphoproteomics of biological fluids also encompass a collection of various protein chemistry techniques, chromatographic separation, and bioinformatics tools.

As a common approach adopted by the research groups engaged in biological fluid phosphoproteomics, the first stage of the research centers on qualitative phosphoproteome discovery. Such qualitative phosphoproteome survey aims to provide description of the catalog of phosphoproteins and the exact assignment of the phosphorylation sites. In addition, bioanalytical workflow optimization is often undertaken at this stage. With some notable exceptions [15,16,17], biofluid phosphoproteome studies executed to date have followed this route to prove feasibility and to generate an initial catalog of phosphoproteins and their sites.

Herein, we review progress in characterizing the phosphoproteome in five major biological fluids: serum/plasma, urine, cerebrospinal fluid (CSF), saliva, and bronchoalveolar lavage fluid (BAL). In the first part we discuss bioanalytical aspects relevant to workflows applied for global phosphoproteome discovery in these biological fluids. In reviewing the bioanalytical strategies, we focus mainly on features pertinent to published biological fluid phosphoproteomics studies, with some discussion of new developments for potential inclusion in future workflows. This review is not intended as a complete presentation of new developments and trends in phosphoproteomics. Instead, when appropriate, we provide references to original research articles or to recent reviews to guide readers interested in more details of a particular sub-topic related to phosphoproteome bioanalytics. In the second part of the review, we present synopses of phosphoproteome studies in individual fluids that have been published to date, and give our opinions on future directions in this field.

## 2. Bioanalytical Strategies

### 2.1. General Workflow Characteristics

**Figure 1:**
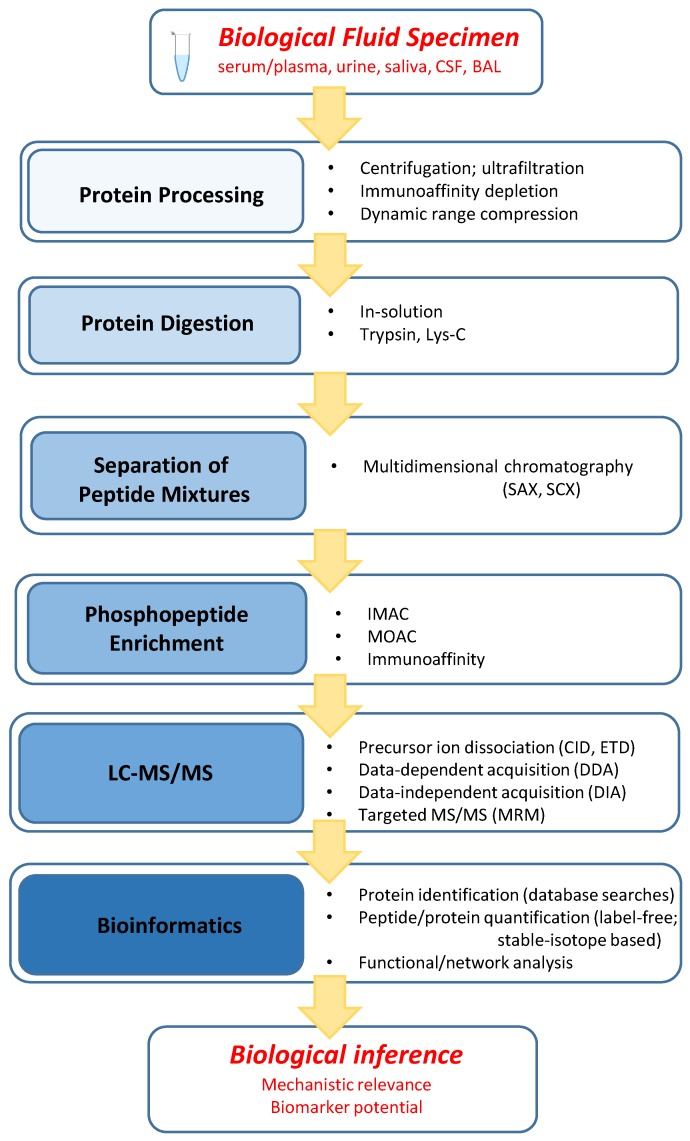
General workflow elements for phosphoproteome characterization in biological fluids. As discussed in the text, there is a variety of options within each element, and not all elements must be part of a chosen workflow. Abbreviations: SAX—strong anion exchange chromatography; SCX—strong cation exchange chromatography; IMAC—immobilized metal ion affinity chromatography; MOAC—metal ion affinity chromatography; CID—collision-induced dissociation; ETD—electron-transfer dissociation; MRM—multiple reaction monitoring.

Phosphoproteome discovery studies in biological fluids to date utilized the so-called bottom-up approach, which denotes a strategy in which information on phosphoproteins present in a biological system and on localization of the phosphosites in these proteins is reassembled from data obtained from direct analysis of proteolytic peptides derived from the constituent proteins. The proteolytic peptides are analyzed with high-end mass spectrometry and bioinformatics tools for amino acid sequence and site assignment information. The general steps in a global phosphoproteome analysis workflow are shown in Figure 1.

Proteomics of biological fluids presents specific challenges. A major issue stems from the wide dynamic range of protein levels and the presence of a small group of high-abundance proteins that constitute a large fraction of the total protein mass [18,19]. These proteins tend to dominate proteomic analyses of plasma/serum and other biological fluids and therefore present a barrier for detection of lower-abundance proteins. For some of the biological fluids, specific other factors such as protein dilution, high content of salts and other interfering components, protease action, etc., need to be taken into account for the design of an effective bioanalytical strategy. Furthermore, the task of phosphoproteome discovery extends beyond determination of protein identities and hence specific peptides bearing the phosphorylation must be probed and the locations of the phosphorylated amino acids must be determined in a comprehensive manner. There is no single, optimum bioanalytical strategy for biofluid phosphoproteomics, and to meet the challenges associated with this research, the phosphoproteome examinations published to date employed various combinations of methods and technologies within the general workflow depicted in Figure 1.

### 2.2. Protein Processing

Specimens of human biological fluids generally require initial processing to eliminate non-protein sample components. As discussed here in Section 3, depending on the type of biological fluid, these contaminants are residual cell debris, salts, lipids and other small molecule components. Furthermore, in some of the fluids such as urine, CSF or BAL, protein is present at very low concentrations. Therefore, the first stage in the processing of raw biological fluid samples for phosphoproteomics commonly involves initial centrifugation to remove particulates and other material, followed by steps to remove contaminants and/or to concentrate the protein analyte. For sample cleanup and protein concentration, ultrafiltration with membrane filters with a specific MW cutoff (typically 3–5 kDa) may be employed; alternatively, protein precipitation with acetone or trichloroacetic acid may be used.

A major consideration associated with (phospho)proteome analyses in serum/plasma and other biological fluids is the wide range of protein amounts. Relative concentrations of serum/plasma proteins span over >10 orders of magnitude. Several protein groups including albumin, serotransferrin, and immunoglobulins comprise more than 95 percent of the total protein mass in plasma [19]. High-abundance proteins or, more precisely the peptides originating from these proteins, are overrepresented in proteome analyses of biological fluids and obscure access to proteins present at lower amounts. To address this challenge, strategies for removal of overabundant proteins and reduction of dynamic range have been developed.

To selectively remove specific high-abundance proteins, antibody-based affinity capture is widely employed. In this “negative chromatography” method, unwanted proteins are bound while the flow-through contains lower-abundance analytes of interest. A number of immunoaffinity columns in various formats have been developed and commercialized. Perhaps the most popular for biological fluid proteomics is termed Multiple Affinity Removal System (MARS) that is available in several versions tailored for removal of a specific number of proteins [20]; for instance MARS Hu-6 is designed to deplete the top six proteins from human biological fluid samples. A drawback associated with depletion of abundant proteins is the loss of proteins that remain bound to the captured carrier proteins such as albumin or that interact nonspecifically with the column [21]. The affinity-bound fraction can be analyzed to probe these proteins at the cost of doubling the number of samples entering the downstream portion of a particular bioanalytical workflow.

Another approach to attenuate the levels of high-abundance proteins utilizes the principle of so-called dynamic range compression [22]. Reagents for this purpose are commercially available under the name ProteoMiner. The method uses combinatorial hexapeptide libraries synthesized on solid support (beads) to provide a pool of affinity ligands, each of them binding a specific protein partner via adsorption. A finite number of molecules from each protein are able to bind to the library to saturate available binding sites for that particular protein; excess protein molecules will remain unbound and be discarded in the flow-through. In this manner, abundant proteins that are present in large excess will be reduced in amount and lower-abundance proteins will be enriched. Thus, in principle this method allows equalization of all proteins within a protein mixture to the same concentration.

### 2.3. Protein Digestion

Trypsin is the most common protease to digest proteins in proteomics and phosphoproteomics. Trypsin cleaves with high specificity at the C-terminal side of Lys and Arg residues, and produces peptides with lengths that are well suited for LC–MS/MS analysis. In phosphoproteome analyses, phosphorylation of serines or threonines in the vicinity of cleavage sites may impair digestion efficiency [23]. A modified digestion methodology that involves pre-digestion of the proteome with endoprotease Lys-C followed by trypsin is one option to enhance reproducibility and the number of identified phosphopeptides [24]. In general, protein digestion may be performed in various formats (in-gel, in-solution, filter-aided sample preparation—FASP [25], etc.), depending on sample type and workflow choice. Specifically for biofluid phosphoproteomics, studies published to date involved in-solution digestion incorporating reduction/alkylation steps prior to protease treatment; urea (at concentrations compatible with protease activity) was used in some applications to aid protein solubilization.

### 2.4. Separation of Peptide Mixtures

For fractionation of complex proteomes, electrophoretic or chromatography approaches are often incorporated to reduce complexity of the peptide mixtures in each LC–MS/MS run. For biological fluid phosphoproteomics, multidimensional chromatography (MDLC) approaches that were employed in some workflows involve strong-cation exchange (SCX) and strong-anion exchange (SAX). The second dimension is then comprised by reversed-phase (RP) in a nanoflow regime, connected online to a mass spectrometer. In SCX or SAX separations, multiple fractions are collected, and each of these fractions undergoes separate LC–MS/MS analysis. Search results for each fraction are concatenated to generate an overall panel. Other modalities may be used in the first dimension of MDLC. For example, RP with high-pH mobile phase has gained popularity in proteomics and phosphoproteomics [26]; this RP–RP approach has not yet been used in biofluid phosphoproteomics. Besides additional variability introduced through the MDLC element, the major issue associated with inclusion of MDLC (or any multidimensional separation) in a bioanalytical workflow is the concern of throughput. The tradeoff between the ability to reach maximum depth of (phospho)proteome coverage vs. the impact on throughput is significant. Workflows that incorporate MDLC suffer this major disadvantage and while they are suitable for studies that intend to profile a small number of samples for initial characterization of a new proteome subset such as the phosphoproteome, they would be exceedingly technically demanding and costly for analyses of large sample cohorts. These drawbacks effectively preclude application of these workflows in clinical biomarker discovery studies. An approach developed recently as an alternative to multidimensional analyte fractionation is single-dimension RP (nanoflow LC–MS/MS) with extended duration of the separation (up to 10 h) [27], and this approach appears to be a valuable option to be considered when extensions of pilot studies to follow up examinations of high number of clinical specimens are being designed [27,28].

### 2.5. Phosphopeptide Enrichment

Upon proteome digestion, the majority of peptides will be non-phosphorylated. This is because upon digestion of a whole proteome from a biological fluid, non-phosphorylated proteins (present in wide range of abundances) will contribute peptides to the overall peptide mixture. Furthermore, phosphorylation is commonly of low abundance and hence a large fraction of a particular protein will be non-phosphorylated, yielding excess of non-phosphorylated peptides compared to their phosphorylated counterparts. Finally, it has been indicated that in LC–MS/MS, a less effective ionization of phosphopeptides may result in unfavorable detection of phosphopeptides vs. non-phosphorylated peptides [29]. For these reasons, to effectively sample the phosphoproteome in the bottom-up approaches, enrichment of phosphorylated species at the peptide level is frequently incorporated in the chosen bioanalytical workflows. Several techniques for phosphopeptide enrichment have been developed, effects of the various formats and experimental conditions on specificity of phosphopeptide isolation and size of phosphopeptide panels have been extensively studied [30], and efforts for further optimization of enrichment strategies for phosphoproteomics are ongoing. Two approaches have emerged as the most prevalent for phosphopeptide enrichment: immobilized metal ion affinity chromatography (IMAC) and metal oxide affinity chromatography (MOAC).

IMAC is an established methodology that utilizes transition metal cations such as Ga^3+^ or Fe^3+^ [30,31,32] immobilized to solid support bearing chelating moieties iminodiacetic acid (IDA) or nitrilotriacetic acid (NTA) to capture peptides bearing negatively charged phosphate groups. Phosphopeptides are loaded onto IMAC at low pH, and after a series of washes elution is achieved at high pH. A variety of modifications in this basic sequence of binding/elution steps have been used with the goal to maximize sensitivity and selectivity [30].

Phosphopeptide capture by MOAC uses the affinity of metal oxides for negatively charged phosphopeptides. The most popular MOAC incorporates titanium dioxide (TiO_2_) as the capture matrix; similarly to IMAC a variety of modifications of the basic experimental protocol have been introduced to optimize performance of the technique. Furthermore, phosphopeptide enrichment workflows with sequential or parallel combination of IMAC and/or TiO_2_ have been employed. Development of new and improved approaches for phosphopeptide enrichment continues [30] and some of these methods could be adopted in biofluid phosphoproteomics, such as sequential elution from IMAC (SIMAC) [33], or affinity enrichment with metal ion-functionalized nanopolymers (PolyMAC) [34]. Collectively, improvements of the different types of phosphopeptide enrichment strategies have been achieved by the phosphoproteomics community but despite these efforts there is no single method that would provide optimum enrichment.

The IMAC and MOAC strategies provide enrichment of pSer-, pThr- and pTyr-containing peptides. For pTyr-specific enrichment, immunoaffinity-based methods have been developed [35], and reagents are commercially available.

Phosphospecies enrichment at the peptide level is the most widely applied strategy. Enrichment may also be performed at the protein level to isolate intact phosphoproteins. This less common approach has been used in the context of serum phosphoproteome discovery [15,16].

Alternatives to IMAC/MOAC chromatography are based on chemical removal of the phosphate moiety and subsequent derivatization with different chemistries that allow enrichment via affinity chromatography [30]. In biological fluid phosphoproteomics, the approach that has been used involved thiol-based derivatization and capture [36].

### 2.6. LC–MS/MS

LC–MS/MS is a key component and the common denominator of (phospho)proteomics workflows. Identification of peptides and proteins in bottom-up approaches is based on data generated by LC–MS/MS analysis of the peptide mixtures produced in proteolytic digestion of the proteome. Reversed-phase chromatography interfaced with high-end tandem mass spectrometers provides separation of the complex analyte mixtures prior to MS and MS/MS. To achieve high sensitivity that is required, the LC configurations commonly feature capillary columns with 75-μm inner diameter and mobile phase flowrates of several hundred nanoliters/min. In an increasing number of LC–MS/MS instrument configurations, the LC is performed in the ultra-high pressure/performance (UPLC) regime, which utilizes sub-2 μm stationary phase particles for major improvements of column efficiencies to achieve high resolution and reproducibility of chromatographic separations.

The peptide analytes in the eluent from nanoLC are introduced into the mass spectrometer and ionized by nanoelectrospray to produce multi-protonated gas-phase molecular ions for MS and MS/MS analysis. The basic goal of the mass spectrometry measurement in the context of (qualitative) phosphopeptide analysis is to determine specific attributes that are then used in subsequent database searches to provide (1) the identity of the proteins present in the sample, and (2) location of the site(s) of phosphorylation in these proteins. Both pieces of information are derived from the mass of the peptide and, most importantly, from the gas-phase dissociation patterns that are diagnostic of the peptide’s amino acid sequence and phosphosite location.

Sensitivity, acquisition speed, and mass measurement accuracy are critical parameters for success of phosphopeptide characterization and site assignment. Several earlier studies of the biological fluid phosphoproteomes were performed with low-resolution LTQ linear ion trap instruments. More recently, as in other sub-fields of MS/MS-based proteomics, hybrid tandem mass spectrometers with configurations of analyzers such as the Orbitrap or time-of-flight (TOF) capable of high resolution/mass accuracy, high data acquisition speed, and increased flexibility in ion-dissociation modes, have been adopted for phosphoproteome discovery in biological fluids. Technological advancements in mass spectrometry instrumentation continue towards maximizing information obtained in a single LC–MS/MS analysis to eliminate the need for upstream fractionation of the analyte mixtures [37].

Gas-phase dissociation of phosphopeptide molecular ions is commonly performed with collision-induced dissociation (CID) to produce sequence-determining product ions of the b- and/or y-series. For protonated phosphopeptide ions, in particular in low-energy CID regime such as in ion trap instruments, an energetically favored fragmentation channel generates a phosphate diagnostic product ion [38]. This ion arises from beta-elimination of the elements of phosphoric acid forming a dehydroalanine. Loss of H_3_PO_4_ (−98 u) from doubly or triply-charged precursor ion (*n* = 2^+^ or 3^+^) generates a non-sequence specific product ion [M + *n*H − H_3_PO_4_]^*n*+^. This product ion can serve as a marker ion, indicating the presence of a phosphorylated peptide. However, oftentimes this product ion dominates the MS/MS spectrum and not enough sequence-determining ions are observed for an unequivocal peptide sequence determination. To address this shortcoming, MS^3^ (i.e., another round of CID on the primary product ion from MS^2^) can be used for confirmation of site assignment on instruments capable of higher-order dissociation. MS^3^ can be triggered when intense primary product ions due to loss of H_3_PO_4_ are detected in the MS^2^ scan. In this manner, the LC–MS/MS datasets contain collections of MS/MS (MS^2^) spectra plus neutral loss-triggered MS^3^ spectra, and both types of data are used for database searches. This strategy, originally developed for analyses on standalone ion trap instruments, was found to be less valuable in LC–MS/MS performed with high-accuracy hybrid instrumentation [39].

An ion activation mode complementary to CID that has been adopted for MS/MS-based phosphoproteome analyses is Electron Transfer Dissociation (ETD) [40]. Upon ETD, dissociation of the activated precursor ions produces product ions of the z- and c-series, thus providing information complementary to low-energy CID where the b- and y-ion series usually dominate. Importantly, phosphorylation, which is labile under CID, is preserved in ETD, and the resulting spectra contain extensive sequence information. To maximize phosphopeptide identification and site localization, both CID and ETD may be incorporated in the phosphoproteomics bioanalytical workflow if an instrument possessing ETD capabilities is available to the investigators.

Another important aspect of LC–MS/MS in (phospho)proteomics concerns the methods of data acquisition. Traditionally, LC–MS/MS of complex proteolytic digests in the bottom-up approach has been performed using data-dependent acquisition (DDA). In DDA, for peptides eluting from LC at any given time, an MS survey scan is acquired to provide information on the masses and intensities of the molecular ions; the MS is then followed by sequential MS/MS scans on a fixed number of precursor ions. This cycle of MS and MS/MS is repeated throughout the whole LC–MS/MS run. Usually, previously interrogated precursor ions are excluded from MS/MS acquisition over a pre-set time window (dynamic exclusion). Real-time selection of molecular ions for MS/MS in each DDA cycle is based on user-set criteria, and is generally biased towards more abundant peptides. Nevertheless, consistent improvements of instrument sensitivity and data acquisition speed have brought enhanced DDA performance [41], and DDA with state-of-the-art mass spectrometry instrumentation continues to be a powerful method for large-scale profiling of complex (phospho)proteomes.

Alternatively to DDA, LC–MS/MS methods have been developed that avoid real-time sampling of individual precursor ions. These data-independent acquisition (DIA) approaches encompass an assortment of different strategies that involve acquisition of MS/MS data independent on precursor ion information [42,43,44,45]. Collectively, these DIA strategies do not involve mass selection of individual precursor ions as the first step in CID. Instead, multiple precursors are selected and dissociated concurrently, either all at once over a single wide *m*/*z* range (in MS^E^ approach [42,44]) or sequentially over smaller windows spanning several tens *m*/*z* (in SWATH method [45]). The MS/MS data generated in these analyses are a composite of CID dissociations of all co-selected precursors and they must be deconvoluted post-acquisition to establish precursor–product ion connectivities. The DIA method applied in biological fluid phosphoproteome discovery is MS^E^, which utilizes a quadrupole-TOF instrument to acquire LC–MS/MS data using alternating collision energy levels to obtain MS (low energy) and MS/MS (high-energy) spectra; the precursor–product ions relationship is reconstructed based on exact overlap of chromatographic profiles for the precursor and the corresponding product ions [42,44].

In most biological fluid phosphoproteomics studies published to date, the focus was on qualitative discovery. Nevertheless, quantitative examinations of the phosphoproteome in serum [16] and urine [17] have been carried out, and the corresponding workflows utilized mass spectrometry-based quantification methods—either label-free or based on stable isotope labeling. In the label-free approach, quantitative information is derived from integrated peak area for the ion chromatogram of the phosphopeptide of interest. Label-free quantification is relatively simple and inexpensive, and it does not involve additional workflow steps. However, multiplexing, i.e., quantification of analytes of interest across multiple conditions in a single LC–MS/MS run, is not possible using the label-free method. Stable isotope labeling pertinent to biofluid phosphoproteomics involves chemical derivatization at the peptide level to introduce stable isotope-containing tags that shift the mass of the labeled phosphopeptide (or a specific product ion) by a known increment. Tags with different combinations of heavy and light isotopes may be used to label peptides in different samples. In this way, peptides in samples from different conditions (such as diseased vs. control) are distinguishable in MS or MS/MS, and relative quantification of the (phospho)peptides of interest in multiple samples may be performed in a single LC–MS/MS analysis. Depending on the composition of the label, quantification may be achieved in MS or in MS/MS (in case of isobaric labeling). One example of a tagging strategy with quantification at the MS level is mTRAQ (mass differential tags for relative and absolute quantification), which involves non-isobaric labeling of primary amines in peptides; mTRAQ has been applied to phosphoproteome quantification in urine [17]. Commercially available tags designed for quantification at the MS/MS level using isobaric labeling include iTRAQ (isobaric tags for relative and absolute quantification) and TMT (tandem mass tags). These approaches utilize isobaric tags whose structure consists of a reporter moiety incorporating a different number/combination of stable heavy isotopes, a balance moiety, and a reactive group that serves to attach the tags to (phospho)peptides after proteolytic digestion. (Phospho)peptides in different samples, when derivatized with these tags have the same precursor ion mass and thus are isolated and dissociated together. However, upon CID, the tagged phosphopeptides produce product ions (so-called reporter ions) that exhibit differences in their m/z. Phosphopeptides originating from different conditions are then quantified based on relative intensities of these reporter ions; amino acid sequence information and phosphosite location is derived from dissociations of the phosphopeptide backbone.

Finally, the LC–MS/MS approaches aimed at global-scale (phospho)proteomics discussed above are complemented by targeted MS/MS. Targeted MS/MS focuses on acquisition of quantitative data for a smaller set of precursor ions selected a priori. A widely used targeted strategy is multiple reaction monitoring (MRM) in which dissociation of a mass-selected precursor to specific product ion(s) (termed transition) is monitored for quantitative measurements [46]. For MRM, the (phospho)peptides to be targeted must be known. Selection of the targets of interest may be based on prior knowledge such as that originating from previous discovery studies, and development of MRM assays has to be undertaken. Today’s LC–MS/MS systems permit high multiplexing of MRM, i.e., MRM data are obtained for many precursors in a single chromatographic run, and MRM acquisition for subsets of precursor–product transitions may be scheduled based on previously established retention times.

### 2.7. Bioinformatics

Bioinformatics elements associated with MS/MS-based phosphoproteome discovery workflows include tools and methods used in global-scale examination of unmodified proteins, with modifications tailored to the purpose of analysis. For identification of phosphopeptides/proteins and for localization of the sites, MS/MS spectra containing series of product ions diagnostic of the peptide amino acid sequence, together with the mass of the corresponding precursor ion, are used in searches of a protein sequence database. Matches between theoretical MS/MS dissociation patterns for peptide sequences in the database and the MS/MS spectra obtained experimentally are generated, and the candidate peptide-spectrum matches are scored and ranked using various scoring algorithms [47]. Target-decoy search strategies are often used to estimate the false discovery rate (FDR) of the dataset [48]. Since phosphorylation increases the mass of the modified amino acid residue by a known differential, putative modification sites can be considered in database searches and the exact location of the phosphosite(s) in a peptide can be determined provided that sufficient information is present in the MS/MS data to allow an unambiguous site assignment. Several software tools have been developed to aid in the task of high-confidence phosphosite assignment [49]. Software packages are also available for quantitative phosphoproteomics with label-free and labeling strategies [50,51]. Beyond phosphopeptide/protein characterization and assignment of phosphorylation sites, additional bioinformatics analyses are frequently applied for functional interpretation of phosphoproteomics findings in the context of molecular networks, pathways and diseases [52]. Finally, information on experimentally determined protein phosphorylation is compiled in the publicly accessible knowledgebase PhosphoSitePlus, which also serves as an interactive resource to facilitate biological interpretation of phosphoproteome data [53].

## 3. Applications to Biofluid Phosphoproteome Characterization

Phosphoproteome discovery studies in serum/plasma, urine, CSF, saliva, and BAL have been carried out. There is a great variety among the individual studies in terms of specimen characteristics, workflow elements utilized, and the size of phosphoproteome panels reported. In addition, the number of specimens analyzed in these phosphoproteome investigations ranged from one [54] to eighty [15]. A snapshot of phosphoproteome studies reported for each biological fluid is shown in Table 1. Details of these studies are presented in Section 3.1 (serum/plasma), Section 3.2 (urine), Section 3.3 (CSF), Section 3.4 (saliva) and Section 3.5 (BAL).

### 3.1. Serum/Plasma

Blood is readily accessible through minimally invasive collection procedures and it is the most widely used biological fluid for diagnostic purposes in routine clinical practice. Blood perfuses all organs and tissues, which contribute to the overall protein composition of this fluid. Thus blood reflects the overall physiology of an individual. Serum is the supernatant fraction remaining after blood clotting and centrifugation. Plasma refers to the fluid obtained after blood collection in the presence of an anti-coagulant and subsequent removal of cells by centrifugation. The total protein concentration in plasma is normally 60–80 mg/mL [61].

Zhou et al. [54] reported on an initial characterization of the phosphoproteome in human serum. To develop a bioanalytical strategy suitable for serum phosphoproteomics, testing of different workflow elements was performed including single and sequential phosphopeptide enrichment strategies, and application of ETD as an additional mode for gas-phase dissociation of phosphopeptide precursor ions. This pilot interrogation was carried out with a serum sample from a single patient with diagnosis of prostate cancer. To define the initial serum phosphoproteome, the serum sample was subjected to immunoaffinity-based depletion of albumin and IgG, and in-solution digested with either trypsin or Lys-C. For enrichment of phosphopeptides in the digests, several approaches were evaluated, including single- or two-round TiO_2_ enrichment, and isolation of phosphotyrosine-containing phosphopeptides with pTyr-specific antibody. For LC–MS/MS analyses of the enriched peptide mixtures, two instruments were employed. LC–MS/MS data were generated with an LTQ-Orbitrap mass spectrometer that used CID for peptide dissociation, and also with an LTQ mass spectrometer using ETD. In total, about 100 unique phosphopeptides were characterized in this qualitative discovery study. From the method development standpoint, two rounds of TiO_2_ enrichment enhanced the number of identified phosphopeptides chiefly due to increased selectivity for isolation of phosphopeptides (i.e., decreased presence of non-phosphorylated peptides as judged by the percentage of matched MS/MS spectra). Of note is the finding that albumin depletion did not improve depth of phosphoproteome coverage compared to analysis of non-depleted sample. Also, the albumin fraction (which was also analyzed) contained a sizable number of phosphopeptides, indicating that a portion of the phosphoproteome is contained in the proteins that remain bound to carrier proteins and that with depletion this information is lost. Interestingly, pTyr-specific enrichment with immunoaffinity did not yield enhanced identification of phosphopeptides bearing this modified residue; two pTyr peptides were found in the set of 100 phosphopeptides after TiO_2_ enrichment, and only one phosphopeptide was detected after treatment with phosphotyrosine-specific antibody. This result could suggest that tyrosine phosphorylation in serum is at low levels and/or that the number of pTyr-proteins in this biological fluid is low. However, in another serum phosphoproteome discovery study involving large sample size, a higher relative proportion of pTyr sites was found (close to 10%) [15].

Carrascal et al. [55] reported on phosphoproteome discovery in human plasma from healthy donors. Three independent experiments were conducted of plasma pools from multiple individuals. The workflow involved immunoaffinity depletion of the seven major plasma proteins via MARS-7; both the MARS-depleted (flow-through) fraction and the protein fraction bound to the MARS immunoaffinity column were analyzed. After tryptic digestion, the peptides were separated by SCX; the phosphopeptides were enriched with TiO_2_. The enriched digests were analyzed on an LTQ ion trap mass spectrometer in the DDA mode, using also the neutral-loss triggered MS^3^. In order to maximize the number of characterized phosphosites and the confidence in phosphopeptide identification and site assignment, the study involved the use of three different search engines (which employ different algorithms) and subsequent cross-comparison of the search results. In total, the study yielded 127 sites in 138 phosphopeptides (at <1% FDR) that mapped to 70 proteins. While the majority of the proteins/sites were discovered (as expected) in the depleted fraction of the plasma proteome, several phosphoproteins were identified in the MARS-bound fraction, thus confirming that complementary—albeit modest—information is obtained in analysis of the MARS-bound proteins. The major functional groups represented in the data included proteins of the complement system and coagulation cascade.

In the serum phosphoproteome examination by Garbis et al. [56], a single pooled serum sample from patients with benign prostate hyperplasia was analyzed. The main focus was on development of a new multidimensional chromatography workflow for analysis of serum proteome. The new workflow applied in this study incorporated several modes of chromatography with different chemistries to provide three dimensions of separation at the protein and peptide level, and this novel workflow was compared with two other approaches that used a different combination of protein/peptide separation methods. In the new workflow, the first dimension of separation at the protein level involved fractionation of the serum proteome by molecular weight with size-exclusion chromatography (SEC). Following in-solution tryptic digestion of the proteins in each SEC fraction, the second and third dimension of separation were performed at the peptide level with off-line zwitterion-hydrophilic interaction chromatography (HILIC) and subsequent nanoUPLC MS/MS. Since the main purpose of the study was evaluation of the novel bioanalytical workflow for serum proteomics, no strategy for enrichment of phosphorylated species was included. With this new workflow encompassing multiple orthogonal chromatographic steps, probing of the serum proteome over wide range of protein abundances was possible and identifications of low-abundance proteins were achieved. In the context of serum phosphoproteome discovery, characterization of a total of 375 phosphopeptides mapping to the same number of proteins was reported. This result indicates that with the multidimensional chromatography workflow evaluated in the study, it is possible to simultaneously probe the serum proteome as well as a sizable number of the serum phosphoproteins. However, as noted in the report no separate effort was done towards validation of phosphosite localization assignment.

In a series of two studies, Jaros et al. focused on characterization of the serum phosphoproteome [15], followed by quantitative assessment of phosphorylation patterns from patients with schizophrenia [16]. For the first phosphoproteome discovery [15], to provide an expanded panel of the serum phosphoproteome, a large set of non-pooled serum samples from 80 donors obtained from two clinical sites was analyzed. The bioanalytical workflow was designed with close attention for future utilization of the workflow and the phosphoprotein/site data. In the chosen workflow, upstream protein processing included depletion with MARS-14 immunoaffinity followed by IMAC enrichment at the protein level. Isolation of intact phosphoproteins rather than phosphopeptides results in the presence of phosphorylated and non-phosphorylated peptides from a particular protein in the tryptic digests of the enriched proteome. This provides protein identification with increased confidence because identifications may be based on two or more peptides, unlike with peptide-based IMAC when (ideally) only phosphorylated peptides are isolated. On the other hand, presence of non-phosphorylated peptides increases peptide mixture complexity with species that are not per se the desired targets of analysis. In this study, mass spectrometry analysis was performed with MS^E^ on a QTOF instrument interfaced with nanoUPLC. After application of strict filtering criteria this large-scale discovery study produced a set of over 5800 phosphopeptides mapping to 502 proteins, which represents a major expansion of the serum phosphoproteome panel compared to the earlier studies. The phosphopeptides reported were found in at least 70% of the 80 samples analyzed. In terms of phosphoproteome characteristics, the panel included a relatively large proportion of pTyr phosphorylation (ca. 10%). This is in contrast to earlier serum phosphoproteome investigations [54], which reported only a limited number of pTyr-containing peptides, despite attempts to specifically target this type of phosphorylation via its selective enrichment. The factors contributing to the difference in the relative proportion of pTyr found in these two examinations would include differences in the bioanalytical workflows and also, importantly, in the number of serum samples analyzed: in the study of Zhou et al., serum from one patient was analyzed and therefore those data reflect the phosphorylation status of a single individual, while results from the Jaros et al’s investigation reflect the combined status of a large cohort. The proportion of pTyr sites found by Jaros et al. is also higher than the ratios reported for cellular phosphoproteomes, in which 90% of phosphorylation occurs on serine residues, and tyrosine phosphorylation generally accounts for less than 1% of the sites [1]. Additional notable characteristics reported for the 502-phosphoprotein dataset is the indication of complex phosphorylation patterns in some serum proteins, as suggested by the large number of phosphopeptides/sites detected in approximately 20% of the proteins. From the functional standpoint, the set included proteins with known association to disease pathophysiologies and also proteins that are known drug targets. Thus, this research, even in its first, qualitative stage, highlighted the importance of serum phosphoproteomics and its potential for future translation into applications in the clinical setting.

Following qualitative phosphoproteome discovery, Jaros et al. [16] interrogated serum proteome and phosphoproteome in cohort of antipsychotic-naïve schizophrenia patients vs. controls (*n* = 20 per group). The bioanalytical workflow was the same as for the discovery study; label-free quantification with MS^E^ was incorporated for differential profiling of protein expression and phosphorylation. For assessment of protein expression changes, IMAC flow-through fraction was also analyzed in addition to IMAC-bound phosphoprotein fraction to allow correlation of protein expression profiles with alterations in protein phosphorylation. Evidence for schizophrenia-associated changes in both relative protein expression levels and in protein phosphorylation was found. In the phosphoprotein panel, 72 phosphoproteins with altered profiles were characterized. Out of this group, 59 phosphoproteins were shown to be altered only in their phosphorylation status without concomitant changes in relative protein abundance. Bioinformatics analysis linked these proteins to molecular networks involved in acute phase response signaling, the complement system, activation of the LXR/RXR nuclear receptors, and several other pathways. Overall, this work provides a nice example of a phosphoproteomics research centered on a specific biological fluid, with initial qualitative phosphoproteome discovery/workflow optimization studies laying foundation for the next research phase focused on quantification of disease-specific phosphorylation changes, to be continued in the future with validation of the phosphoprotein biomarker candidates.

### 3.2. Urine

Urine is the product of filtration of blood by the kidney and contains proteins from this filtration as well as proteins originating from the kidney itself. Thus, protein composition of urine reflect the systemic physiology and also status of the kidney and the urogenital tract. Collection of urine is a simple, non-invasive procedure. Protein concentration in normal urine is very low (<0.1 mg/mL [62]) but a large volume of urine can be obtained easily.

In a study with a combined focus on urinary proteome and phosphoproteome, Li et al. [57] examined urine samples from three healthy donors. Processing of the samples involved acetone precipitation of urinary proteins followed by tryptic digestion, and the peptide digests (without any phosphopeptide-specific affinity capture step) were subjected to two different multidimensional chromatography workflows. The first approach (which the authors termed Integrated Multidimensional Liquid Chromatography, IMDL) used a bi-sectional column with SCX and RP packing, interfaced online with an LTQ-Orbitrap mass spectrometer; elution from the SCX was accomplished with a low-to-high pH gradient. The second multidimensional “Yin-yang” chromatography employed an off-line configuration with parallel separation by SCX and SAX (of peptide flow-through fraction from SCX), and (RP)LC–MS/MS of all fractions collected in both ion-exchange separations. In their previous work [63], the authors showed the benefits of the inclusion of SAX to enhance characterization of phosphopeptides (and other acidic peptides) that do not bind to SCX under the initial low-pH conditions. For the urinary phosphoproteome component of the study, the examination yielded 45 unique phosphopeptides containing 59 phosphosites mostly on serine residues. The peptides mapped to a total of 31 proteins; the majority of proteins contained 1–2 phosphosites, with the exception of osteopontin for which 18 sites were characterized. Hyperphosphorylation of osteopontin was highlighted in context of possible future functional follow-up of these phosphoproteins in relationship to kidney stone formation.

Zheng et al. [17] reported on quantitative profiling of the urine phosphoproteome in healthy women before and after delivery as a foundation for the discovery of biomarkers for pregnancy-related pathophysiological conditions. Phosphoproteomics was undertaken in addition to qualitative large-scale mapping of the urinary proteome. Urine samples (pools from multiple individual donors) were desalted and concentrated with ultrafiltration, and the urinary proteins were precipitated with trichloroacetic acid. The proteins were digested in solution with trypsin, the peptides were labeled with the non-isobaric mTRAQ reagent to enable MS-based quantification, and phosphopeptides were enriched with TiO_2_. In lieu of multidimensional separations, a “single-run” LC–MS/MS strategy was selected in which ultra-long (10 h) nanoUPLC separations with shallow mobile phase gradient, interfaced with an LTQ-Orbitrap, were performed [27]. In total, 130 unique phosphopeptides with 222 sites, mapping to 105 phosphoproteins were characterized. Seventy percent of the phosphosites were accounted for by phosphoserines; 22% at phosphothreonines, and 8% of phosphotyrosine residues. Sixteen phosphoproteins were found to be differentially regulated. Overall, the panel mapped is nearly four-fold larger compared to Li et al. [57] (which used multidimensional chromatography without phosphospecific enrichment to allow simultaneous protein expression/protein phosphorylation comparisons). Therefore, the relatively simple workflow applied in this study yields urinary phosphoproteome panel of a substantial size. In contrast to multidimensional workflows where a number of fractions per sample need to be analyzed (LC–MS/MS analysis of each fraction typically involves ca. 2 h-long LC gradient), the simplicity of downstream (phospho)peptide analysis makes the “single-run” long-gradient workflow practical for applications to urinary phosphoproteomics examinations of large numbers of samples to ensure a reasonable throughput.

Zhao et al. [58] investigated human urine with the objective to map the urinary phosphoproteome, and examine the effect of protein phosphatases present in urine on the characteristics of renal cell carcinoma-derived phosphoproteome to identify phosphatase-stable phosphoproteins/sites for potential exploration as disease biomarkers. Individual and pooled samples from three healthy volunteers were analyzed. Selected aliquots of the urine samples were incubated with A498 kidney carcinoma cell lysates to simulate action of urinary phosphatases on the phosphoproteins released from cells. Proteins in all urine samples were precipitated with acetone and in-solution digested with trypsin. Titanium dioxide enrichment of phosphopeptides was employed, and the enriched digests were analyzed by nanoLC–MS/MS on an LTQ-Orbitrap instrument. In analyses of untreated urine, a total of 106 phosphosites in 64 phosphoproteins were characterized in samples from the three individuals (in three technical replicates per sample). Close to 92% of the sites were localized on serines. Out of this set of phosphoproteins which represent the urinary phosphoproteome probed in this study, only eight phosphoproteins were common to all three samples, underscoring the degree of biological variability associated with human urinary phosphoproteome, and the limitations in examining small number of biological replicates.

### 3.3. Cerebrospinal Fluid

Cerebrospinal fluid (CSF) is in direct contact with the entire surface of the central nervous system. Because of its proximity to the brain and the spinal cord, molecular constituents in CSF reflect the status of these structures. CSF is most commonly collected via lumbar puncture, which is an invasive procedure [64]. Normally only a small amount of protein is present in CSF, and protein concentration in CSF (ca. 0.5 mg/mL) is 1% of that of serum [24,65].

In examination of CSF by Bahl et al. [24], pooled samples from multiple individuals were analyzed with the goal to optimize the bioanalytical workflow, with particular emphasis on the processing of CSF, protein digestion, and phosphopeptide enrichment, and to produce the initial description of the phosphoproteome in this biological fluid. Because of the low protein concentration in CSF, spin filters with 3 kDa cutoff were employed to concentrate CSF proteins. For digestion, the authors reported that multiprotease combination of Lys-C followed by trypsin was used to enhance digestion efficiency to result in improved coverage of the CSF phosphoproteome. Deglycosylation step with with PNGase F was included to remove sialic acid-containing glycans that may interfere with TiO_2_ capture of CSF-derived phosphopeptides [66]. Based on LC–MS/MS performed with an LTQ-Orbitrap mass spectrometer using multistage activation (pseudo-MS^3^), phosphopeptides mapping to 44 phosphoproteins were identified in this study. Thirty-eight of the proteins contained a total of 56 phosphosites that were novel at the time of publication. Some of these were new sites discovered in known phosphoproteins such as secretogranin 1; in addition, a number of proteins were described for the first time to exist in their phosphorylated forms.

### 3.4. Saliva

Saliva is a clear fluid that serves to aid food processing, to maintain health of the oral cavity, and in other functions [67,68,69]. Saliva specimens are easy to collect by non-invasive means. Whole saliva is composed of proteins and other biomolecules originating from major and minor salivary glands and from gingival crevicular fluid; in addition to proteins intrinsic to the salivary glands, saliva also contains serum components. Composition of saliva is influenced by many factors, such as circadian rhythm, rate of flow, stress, and numerous other factors which may contribute to variability in saliva composition among individuals and also within the same individual.

Salih et al. [36] investigated whole saliva to characterize on large-scale the salivary phosphoproteome as an initial phase of a long-term direction towards developing saliva-based biomarkers for diagnosis for oral and systemic disease. Pooled samples of whole saliva collected from five individuals were used in multiple analyses. The samples were centrifuged and the proteome was digested with trypsin. To enrich for phosphorylated peptides, the bioanalytical workflow used chemical derivatization of phosphoserine and phosphothreonine residues by a thiol reagent (dithiothreitol, DTT); this reaction involved base-catalyzed beta-elimination of the phosphate group followed by reaction of the resultant dehydroalanine with DTT. The DTT-derivatized peptides were isolated with thiol interchange chromatography using Sepharose 4B glutathione-pyridyl disulfide. The enriched digests were analyzed by nanoLC–MS/MS with an LTQ linear ion trap mass spectrometer. Upon CID, in contrast to regular phosphopeptide molecular ions that undergo facile loss of phosphoric acid, the DTT-derivatized phosphopeptides showed enhanced sequence-specific product ions in their MS/MS spectra. Overall, the initial catalog of phosphoproteins obtained in this study of whole saliva encompassed 65 proteins, classified as originating from multiple cellular sources.

To expand the inventory of whole saliva phosphoproteins, Stone et al. [59] analyzed the phosphoproteome in pooled samples procured from healthy donors. Processing of the whole saliva samples involved centrifugation followed by treatment with ProteoMiner hexapeptide library beads to reduce the amounts of high-abundance proteins while simultaneously capturing and concentrating lower-abundance proteins. Non-treated samples were also analyzed. SDS-PAGE in combination with ProQ Diamond phospho-specific staining was used to obtain an initial profile of the whole saliva phosphoproteome. For MS/MS-based phosphoproteomics, the Proteominer-treated and untreated samples were digested in solution with trypsin. The peptides in the digests were separated by off-line SCX chromatography and phosphopeptides in each SCX fraction were enriched with IMAC. LC–MS/MS was performed with an LTQ-Orbitrap mass spectrometer; to maximize phosphopeptide detection, MS/MS incorporated CID and ETD. Protein sequence database searches with the LC–MS/MS datasets considered semitryptic specificity among the search parameters to enable characterization of salivary phosphoproteins that underwent post-translational processing. Because initial searches gave inconclusive output for tyrosine-phosphorylated peptides, the searches were repeated with only pSer and pThr considered as variable modifications (together with oxidation of methionine) to achieve <2% FDR. With the chosen bioanalytical and informatics format, a total of 217 distinct phosphopeptides were characterized in 85 phosphoproteins. The effect of dynamic range compression (ProteoMiner treatment) was not compelling in this particular application; 17% of the phosphopeptides in the characterized set were unique to the ProteoMiner-processed samples. Similarly, ETD-based LC–MS/MS provided unique characterizations of 22 phosphopeptides (10% of the total panel) and corroboration of additional 30 phosphopeptides that were also detected based on CID MS/MS spectra. Comparison of cellular distribution of the characterized phosphoproteins to that of salivary glycoprotein panel generated by the same group in a separate study revealed differences in the distribution of the phospho- vs. glycoproteins. One third of the characterized phosphoproteins were from cytoplasm while the majority of the glycoproteins were categorized as extracellular or localized to plasma membrane. Alignment of the peptide sequences flanking the characterized phosphosites to identify consensus sequences showed diversity of kinase recognition motifs suggesting a variety of kinases potentially acting on the salivary phosphoproteome.

### 3.5. Bronchoalveolar Lavage Fluid

In contrast to the other biological fluids discussed herein, BAL is not produced naturally. Specimens of BAL are procured via fiberoptic bronchoscopy as washout of the epithelial lining of the lung with a saline vehicle. The procedure to obtain BAL is invasive but generally well tolerated [70]. The protein composition in BAL includes proteins released locally by the airway epithelium and other resident cell types, and also serum proteins diffusing across the air–blood barrier [71,72]. Presence of overabundant serum proteins, high salt content, and analyte dilution (tens of mL of BAL are typically collected) necessitate inclusion of analyte concentration/salt removal steps in the upstream sample processing. Depletion of overabundant proteins is also to be considered in bioanalytical workflows for BAL (phospho)proteomics [73].

Giorgianni et al. [60] completed pilot interrogation of the BAL phosphoproteome to generate an initial phosphoproteome catalog of this biological fluid. This study involved analyses of two pools of 3–7 individual human BAL samples in two independent experiments; BAL specimens were from individuals without diagnosis of lung cancer or chronic obstructive pulmonary disease. BAL samples were centrifuged to remove cell debris, followed by desalting/protein concentration via ultrafiltration. High-abundance proteins were depleted with Hu-6 MARS immunoaffinity chromatography, in-solution digested with trypsin, and enriched with IMAC. LC–MS/MS was performed with an LTQ ion trap mass spectrometer. With this bioanalytical workflow, 26 phosphosites were characterized in 36 phosphopeptides mapping to 21 proteins. The MARS-bound portion of the phosphoproteome was not analyzed. Phosphorylated serine residues comprised 92% of the phosphosites characterized. Analogously to pilot phosphoproteome discoveries in other biological fluids, large variability in the phosphoproteins between the two sample pools was observed, with only five phosphoproteins (24%) detected in both samples. Based on information collected from the Human Proteome Atlas (HPA), the phosphoproteins were expressed in the lung and in other tissues, reflecting the diverse origin of BAL proteins. This initial examination of BAL demonstrated that phosphoproteome discovery in BAL is feasible, and provided the first map of BAL phosphoproteins, intended as a foundation for future biomarker discovery studies.

## 4. Concluding Remarks

Phosphoproteomics interrogation of major biological fluids provides an attractive opportunity to gain expanded, unique scientific knowledge complementary to global-scale profiling of protein expression. This unique knowledge on protein phosphorylation holds the promise of new mechanistic insights into protein function in health and disease, and the potential to be translated into clinical applications for improved patient care outcomes. Despite the potential for major impact, research on the phosphoproteome in biological fluids occupies a small niche in proteomics. There are a handful of groups who have published in this arena, and the pace of moving beyond initial phosphoproteome discovery has generally been slow. We believe that it is time to regain the momentum, and we hope that this review will spark renewed interest in biological fluid phosphoproteomics.

From the technical standpoint, there is a huge variety of options for essentially every element of contemporary bioanalytical strategies for phosphoproteomics, from upstream sample processing, to separations of the analytes at the protein and/or peptide level, enrichment strategies, LC-MS/MS configurations and data acquisition modes, and final bioinformatics analyses. The plethora of bioanalytical workflows used to date in the field reviewed herein reflects these realities. Looking into the future, as the field progresses towards the examination of large sample cohorts, sample throughput and method robustness will become prime considerations even if counterbalanced by relatively moderate phosphoproteome coverage. Therefore, workflows that rely on “single-shot” analyses with extended LC gradients and/or with latest-generation high-performance mass spectrometers will likely prevail over approaches involving multidimensional separations. For quantification, multiplexed MRM is a prospective option, targeting panels of phosphopeptides chosen to be pursued as e.g., biomarker candidates based on phosphoproteome discovery and/or pilot comparative phosphoproteome profiling. DIA strategies are to be watched for further developments in computational data analysis and other aspects; it remains to be seen if these methods fulfill their promise and are broadly adopted in the coming years.

As discussed in this review, the initial discovery phase of the phosphoproteomes in the five major biological fluids has been carried out. The size of the phosphoproteome panels described varies considerably from ca. 20 phosphoproteins mapped in BAL to 500 phosphoproteins characterized in plasma. There is certainly opportunity for continued discovery to deepen the phosphoproteome coverage in the fluids, and research in this direction should continue. However, the sets of phosphoproteins and exact sites of phosphorylation assigned in these proteins already represent a wealth of discoveries to be pursued further. Thus the foundation has been laid and research studies building on this foundation are needed to capitalize on and expand the initial findings to move the field forward.

Although qualitative discoveries have uncovered phosphoproteins that are known to be disease-relevant, transition from qualitative discovery to the quantitative assessment of phosphorylation changes in a carefully defined context needs to be pursued more vigorously. Phosphorylation status of a biological fluid proteome reflect the health/disease status of distant tissue or proximal organs. For biomarker discovery and development, biofluid phosphoproteomics should take into account the (hard) lessons learned from the decade-and-a-half of biomarker proteomics efforts. Close attention should be paid to issues such as collection of high-quality biological specimens and proper study design. Continued innovations in analytical technologies and informatics tools are anticipated and these should be harnessed for improved workflows, together with efforts for standardization to reduce variability across workflows and laboratories. Active collaboration between basic and clinical scientists is necessary to define a clinically meaningful question for which biomarker(s) is intended, and to drive the research in that direction, which extends far beyond publications of phosphoproteins lists or putative biomarker panels. Finally, to sustain this drive in the long term, allocation of sufficient resources is required. While we must operate within the current budget realities, a research route that ends with dissemination of pilot phosphoproteome findings would be an opportunity missed. Collectively, investments to support longer-term translational endeavors, commitment of expert teams to these endeavors, and technological advancements are critical factors that will influence the future of biological fluid phosphoproteomics research and shape its ultimate impact.

## Figures and Tables

**Table 1:** Summary of phosphoproteome discovery studies in five biological fluids: serum/plasma, urine, CSF, saliva, and BAL. Details of these studies are presented in the text (Section 3.1, Section 3.2, Section 3.3, Section 3.4 and Section 3.5). MDLC: multidimensional chromatography; CID: collision-induced dissociation; ETD: electron-transfer dissociation; DDA: data-dependent acquisition; DIA: data-independent acquisition.

Fluid Studied	Disease or Condition	Protein Depletion	MDLC	Phospho Enrichment	CID/ETD	DDA/DIA	Phosphoproteome Panel Reported	Reference
Serum	Prostate Cancer	Y	N	Y	Y/Y	DDA (qual.)	~100 phosphopeptides	[54]
Plasma	Normal	Y	Y	Y	Y/N	DDA (qual.)	138 phosphopeptides/127 sites in 70 proteins	[55]
Serum	Benign Prostate Hyperplasia	N	Y	Y	Y/N	DDA (qual.)	375 phosphopeptides in 375 proteins	[56]
Serum	N/A	Y	N	Y (at protein level)	Y/N	DIA (qual.)	5800 phosphopeptides in 502 proteins	[15]
Serum	Schizophrenia vs. Control	Y	N	Y (at protein level)	Y/N	DIA (quant.)	59 altered phosphoproteins	[16]
Urine	Normal	N	Y	N	Y/N	DDA (qual.)	45 phosphopeptides/59 sites in 31 proteins	[57]
Urine	Pregnancy (Before/after delivery)	N	N	Y	Y/N	DDA (quant.)	130 phosphopeptides/222 sites in 105 proteins; 16 altered phosphoproteins	[17]
Urine	Normal	N	N	Y	Y/N	DDA (qual.)	106 phosphosites in 64 proteins	[58]
CSF	Suspected Neurological Disorder	N	N	Y	Y/N	DDA (qual.)	44 phosphoproteins (include 56 novel sites)	[24]
Saliva	Normal	N	N	Y (derivatization)	Y/N	DDA (qual.)	65 phosphoproteins	[36]
Saliva	Normal	Y	Y	Y	Y/Y	DDA (qual.)	217 phosphopeptides in 85 phosphoproteins	[59]
BAL	N/A (*not* Lung Cancer or COPD)	Y	N	Y	Y/N	DDA (qual.)	36 phosphopeptides/26 sites in 21 proteins	[60]

## Abbreviations

The following abbreviations are used in this manuscript:

BAL	bronchoalveolar lavage fluid
CID	collision-induced dissociation
CSF	cerebrospinal fluid
DIA	data-independent acquisition
DDA	data-dependent acquisition
ETD	electron-transfer dissociation
IMAC	immobilized metal ion affinity chromatography
iTRAQ	isobaric tag for relative and absolute quantification
MARS	Multiple Affinity Removal System
MDLC	multidimensional liquid chromatography
MRM	multiple reaction monitoring
MOAC	metal oxide affinity chromatography
mTRAQ	mass differential tag for relative and absolute quantification
RP	reversed-phase
SAX	strong anion exchange
SCX	strong cation exchange
TMT	tandem mass tag
TOF	time-of-flight
UPLC	ultra-high pressure chromatography
